# Percutaneous Vertebroplasty as the Treatment of Choice for Multiple Adjacent Lumbar Atypical Haemangiomas: A Case Report

**DOI:** 10.7759/cureus.58171

**Published:** 2024-04-13

**Authors:** Davide De Los Rios, Cristiana Germano, Sergio Corvino, Antonio Bocchetti, Giuseppe Corazzelli

**Affiliations:** 1 Department of Medicine, Università degli Studi di Napoli "Federico II", Naples, ITA; 2 Maxillofacial Surgery Unit, Department of Neurosciences, Reproductive and Odontostomatological Sciences, Università degli Studi di Napoli "Federico II", Naples, ITA; 3 Neurosurgery Division, Department of Neurosciences, Reproductive and Odontostomatological Sciences, Università degli Studi di Napoli "Federico II", Napoli, ITA; 4 Neurosurgery Unit, Ospedale Santa Maria delle Grazie, Pozzuoli, ITA; 5 Neurosurgery Division, Department of Neurosciences, Reproductive and Odontostomatological Sciences, Università degli Studi di Napoli "Federico II", Naples, ITA

**Keywords:** atypical adjacent haemangiomas, polka-dot sign, polymethylmethacrylate, percutaneous vertebroplasty, vertebral haemangioma

## Abstract

Atypical vertebral haemangiomas (VHs) are uncommon lesions that cause lumbar pain and motor symptoms. Current management mainly relies on radiotherapy, surgical spine decompression, or percutaneous techniques. We describe a unique case of a patient with two adjacent lumbar VHs and an underlying lumbar fracture which was treated only by percutaneous vertebroplasty (PV). The non-invasive technique relieved the patient’s pain and did not affect column stability. PV may be considered an amenable pain-relieving treatment for adjacent atypical VHs in selected patients.

## Introduction

Atypical Vertebral haemangiomas (VHs) are rare tumors of the lumbar spine. Lumbar pain and motor symptoms are common manifestations [1]. These are vertebral angiomas with atypical features, such as an adipose signal, a tendency to the involvement of paravertebral tissues, the atypical signal on Computed Tomography (CT) and Magnetic Resonance Imaging (MRI), and a tendency to destabilize the spine. Spine CT is the radiological examination that allows diagnosis. It can reveal “Polka-Dot” and “Honeycomb” patterns and the trabeculated structure of the spongiosa of the vertebral body. In addition, magnetic resonance imaging (MRI) may also show the degree of soft tissue extension, fat content, spinal compression, and specific aggressive behavior indicators [2]. We present the case of a patient with two adjacent lumbar atypical VHs, both occupying nearly all the vertebral somas and threatening column stability, treated by percutaneous vertebroplasty (PV). This peculiar and treacherous condition benefited from PV, not requiring surgical decompression. In the literature, there is no consensus treatment for this condition. Management of atypical VHs is usually consistent with percutaneous preoperative embolization of the afference, surgical decompression of the level, or radiation therapy [3]. Our study might provide substantial evidence for correctly managing these rare conditions.

## Case presentation

**Figure 1:**
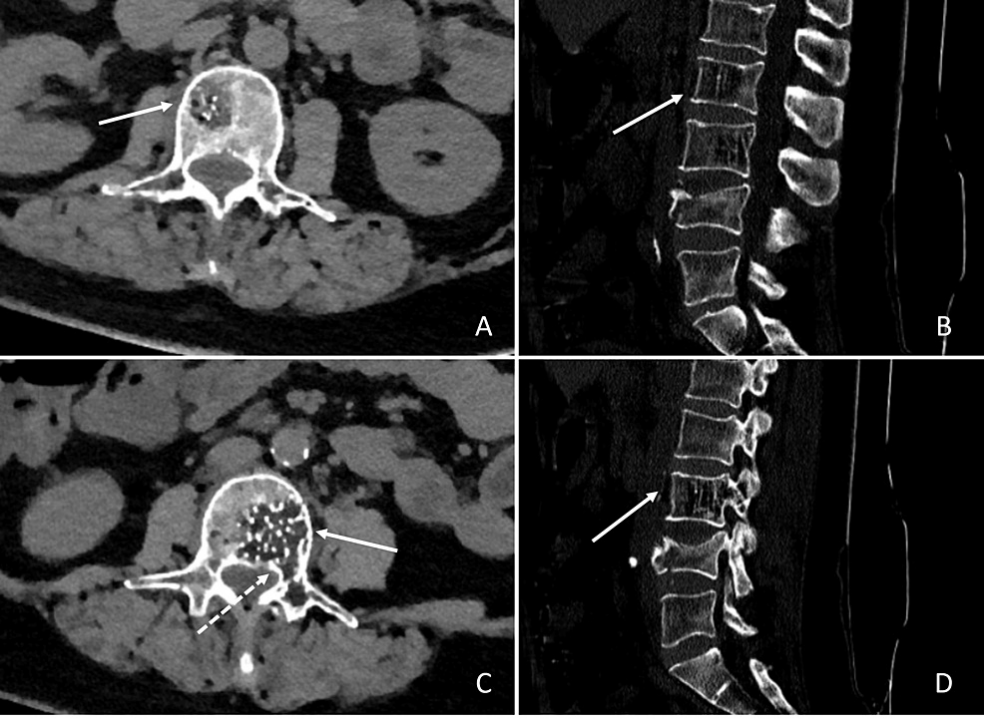
CT images of the patient There was evidence of lumbar adjacent atypical vertebral haemangiomas in L2 and L3 bodies. A. Axial image of L2. Microfractures were evident, as well as trabeculate structure (white arrow). B. Sagittal image of the lumbar column, paramedian right. C. Axial image of L3. The trabeculate pattern was evident on this scan (white arrow), as well as the involvement of the left peduncle (dotted white arrow). D. Sagittal image of the lumbar column, paramedian left. The “Honeycomb” structure was evident in the correspondence of the soma of L3 (white arrow).

A 65-year-old woman came to our attention complaining about low back pain. In history, the patient reported no notable pathologies. Neurological and physical examination at admission was negative. A lumbar computed tomography (CT) showed two atypical lumbar haemangiomas at L2 and L3 and an osteoporotic L4 soma fracture (osteoporotic fracture type 4). At L2 and L3 soma, there was evidence of the characteristic “honeycomb” pattern, the trabeculate structure, and the “polka-dot” sign in both segments, suggesting two adjacent atypical VHs [1]. Furthermore, the lesion on the vertebral soma of L3 extended up to the segment’s left peduncle and transverse process, configuring a potential threat to lumbar stability (Figure 1).

**Figure 2:**
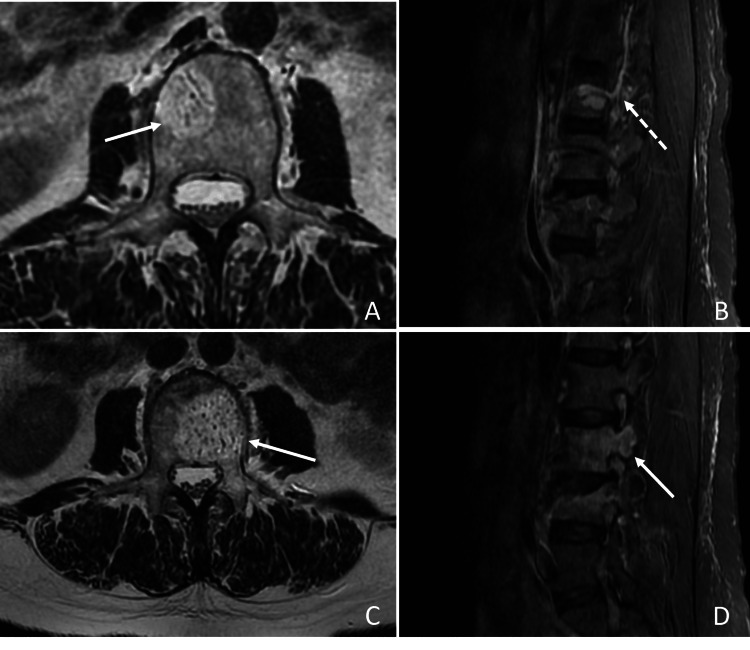
Magnetic resonance imaging (MRI) of the lumbar spine A and C, T2-weighted axial sequences; B and D, STIR (Short Tau Inversion Recovery) sagittal sequences. The MRI confirmed the presence of two adjacent atypical haemangiomatous neoformations at L2 and L3 without invasion or compression of the spinal canal. A. T2-2 axial sequence of L2. The fat-suppressed sequence demonstrated an important fat component within the neoformation (white arrow). B. STIR sagittal sequence of the lumbar spine, paramedian right. The right L2 pedicle involvement was found (hatched arrow). C. T2-w axial sequence of L3. Extending and invading the left peduncle (white arrow) was a critical fat component. D. STIR sagittal sequence of the lumbar spine, paramedian left. The left L3 peduncle was hyperintensified due to the extension of the haemangioma (white arrow). The preoperative MRI showed no ballooning, cortex lysis, or extraosseous soft tissue extension.

Subsequent magnetic resonance imaging (MRI) of the lumbar spine revealed the presence of an atypical vertebral haemangioma in the soma of L2, having peculiar paraspinal vascular afferents, whereas the L3 atypical haemangioma showed a huge fatty component (Figure 2).

**Figure 3:**
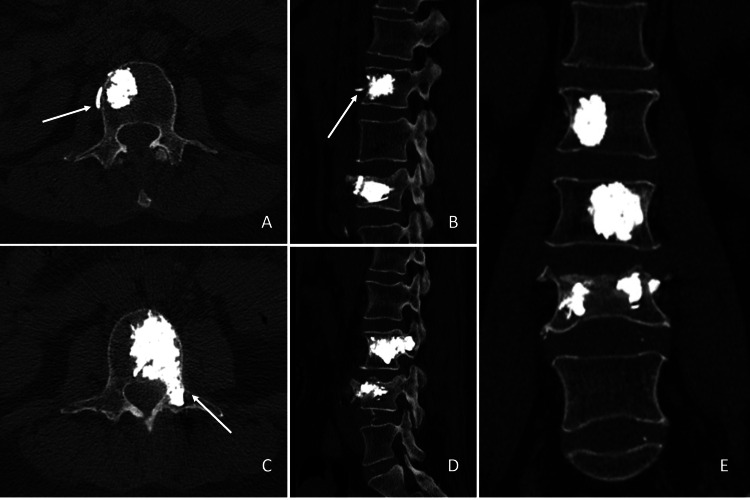
Postoperative computed tomography of the lumbar spine A and C, axial images; B and D, sagittal images; E, coronal image. On the first postoperative day, computed tomography of the lumbar spine confirmed the correct positioning of the cementing material in both vertebral bodies and the left L3 peduncle. A. Axial image of L2. B. Sagittal image of the lumbar spine, paramedian right. The anatomical vessel distribution of the hyperdense PMMA allows appreciation of the obliteration of the vascular afferents (white arrow). C. Axial image of L3. Hardening of the left peduncle was achieved (white arrow). D. Sagittal image of the lumbar spine, paramedian left. E. Coronal image of the lumbar spine.

Due to the potential future instability of the segments, blended with the patient's coherent osteogenic pain, the indication was placed for a surgical procedure. The lower back pain might be related to the fracture, deserving an indication for surgery. The choice to cement the two VHs is also derived from the prediction that a cemented vertebra, over time, could lead to fracture of the two somas above, already diffusely involved by VHs. Lumbar arthrodesis was declined in favor of the PV of both the VHs and the fracture. The rationale for the choice lay in the minimally invasive nature of vertebroplasty compared to the spinal stabilization procedure. Percutaneous vertebroplasty of both the haemangiomatous soma and the fractured segment was performed. In detail, using a right percutaneous mono-pedicular approach, we proceeded to inject polymethylmethacrylate (PMMA) in the right half of the soma of L2, obliterating paraspinal vascular afferents; the same was made for the L3 vertebral body, including left pediculoplasty. Finally, we performed percutaneous vertebral augmentation with an expandable SpineJack system and bi-pedicular vertebroplasty in the L4 vertebral body. The postoperative lumbar CT confirmed the correct positioning of the cementing material in the vertebral bodies and the obliteration of both vertebral haemangiomas (Figure 3).

The postoperative course was uneventful, and the lower back pain was entirely resolved. Three years after surgery, the patient presented neurologically intact and resolved the lower back pain. The complex management of this patient is worthy of attention as it is a complex spinal administration aimed at the patient's well-being both concerning the present but also the prospects of a future need for instrumentation of one cemented vertebra and two vertebrae involved by atypical VHs.

## Discussion

In clinical practice, the spine exhibits multiple rare pathologies [4,5], among them are atypical VHs [1]. These rare lesions differ from classic VHs, which are a common incidental radiological finding, and account for 2% to 3% of all spinal tumors. A study on a large autopsy series recently estimated an overall incidence of 11% in the adult population [6]. The most common location of atypical VHs is the vertebral body of the thoracic spine, but they may frequently localize at the lumbar level [7]. Although most VHs do not need treatment or observation, some may exhibit aggressive behaviors and features. This was an interesting case of two adjacent vascular lesions. Features of atypical VHs are rapid growth, extension far beyond the vertebral body, invasion of the paravertebral or epidural space, and compression of the spinal cord or nerve roots [8,9]. In our case, the vast somatic involvement, the peduncular extension, and the visible vascular afference were elements threatening potential future lumbar instability. Furthermore, the two atypical VHs were in adjacent levels. Moreover, their extent, fat content, and vascular afferents strongly suggest increasing their size over time. Finally, the patient had reported an osteoporotic fracture deserving of surgical indication at the adjacent level. Depending on the need for stabilization of the fracture, all three vertebrae were cemented. The patient benefited from this treatment and did not require any further instrumental treatment of the lumbar spine.

CT represents the most suitable imaging modality, consistent with the literature; the patient’s admission CT showed a “honeycomb” structure and the characteristic “polka-dot” sign, typical findings of atypical VHs [10]. Interestingly, the MRI showed the T1-weighted hyperintensity due to the fatty tissue and T2 hyperintensity due to the water content within the VHs. Moreover, preoperative imaging studies also revealed a remarkable paraspinal vascular afference at the right side of the L2 body. These were described as aggressive behavior indicators [1]. 

In a small subset of patients, atypical VH growth may lead to circumferential vertebral involvement, including anterior and posterior elements [8]. Other CT findings include ballooning or lysis of the cortex or extraosseous soft tissue extension [8]. Such indexes of vertebral instability were absent at the admission time. Furthermore, the two lesions at L2 and L3 did not cause compression on the cord or instability of the lumbar spine. Therefore, there was no need for spinal decompression or lumbar stabilization. On the other hand, there was a rationale for radically treating these lesions, as volumetric growth over time was almost assured, as was pain, given the unambiguous scientific evidence in the literature [1,2,6]. The adjacency to a fractured soma was another element suggesting intervention. Therefore, based on the radiological features and clinical symptomatology, we decided to perform PV of L2, L3, and L4 bodies after agreeing with the patient. Recent studies slightly demonstrated PV to relieve pain in patients with vertebral atypical haemangiomas [11]. The mechanism by which vertebroplasty results in pain relief is still unknown. Some studies report it might be related to stabilizing microfractures within the soma and preventing further compression or deformity and a PMMA-induced chemical ablation of pain-sensitive nerve endings within the vertebral body [12]. A recent study on the appropriate management of symptomatic VHs found multiple lesions in 12% of cases [12]. Due to the rarity of this condition, and the multidisciplinary interest in the pathology, few studies report the proper management of multiple levels of atypical VHs. Furthermore, none of the examined studies reported cases of multiple VHs at contiguous levels.

There is still no consensus on the best treatment strategy for symptomatic VHs; thus, the patient’s symptoms and imaging features should route the correct management. Many approaches have been experimented with in time, from conservative medical therapy to radiotherapy, surgical decompression, and percutaneous techniques (vertebroplasty, trans-arterial embolization, direct intralesional ethanol injection) or a combination of these [13,14].

Surgical decompression and lumbar stabilization are choice treatments in case of canal involvement and neurological deficit or column instability [15]. As well these treatments lead to muscular denervation, subsequent hypotrophy, and functional impairment [9]. From an invasiveness point of view, PV should be better evaluated as a surgical option in selected cases due to the patient’s better tolerance and postoperative pain relief. Lastly, the sclerotizing effect of PMMA on the vascular tissue of the angiomatous lesions has been hypothesized, along with the welding of vertebral microfractures.

This was the first case of multiple atypical VHs in adjacent lumbar segments. PMMA can provide internal consolidation of trabecular microfractures of the spongious vertebral bone and ablation of the pain amyelinic somatic terminations. Notably, percutaneous vertebroplasty also has a lower risk of complications. It provides an immediate antalgic effect, allows for a quick recovery of mobility, and results in durable somatic hardening and stability [11].

## Conclusions

This was an anecdotal case of adjacent painful lesions. PV is a minimally invasive technique that is an effective strategy for atypical VHs. In our case, it resulted in complete clinical relief and, over time, no need for vertebral arthrodesis of a severe osteoporotic fracture. Research should shift from the most invasive techniques toward less invasive surgical procedures intelligently chosen for selected patients based on predicting future junctional instability.
